# Using psychological theory to understand the clinical management of type 2 diabetes in Primary Care: a comparison across two European countries

**DOI:** 10.1186/1472-6963-9-140

**Published:** 2009-08-05

**Authors:** Susan Hrisos, Martin P Eccles, Jill J Francis, Marije Bosch, Rob Dijkstra, Marie Johnston, Richard Grol, Eileen FS Kaner, Ian N Steen

**Affiliations:** 1 Institute of Health and Society, Newcastle University, Newcastle Upon Tyne, UK; 2 Health Services Research Unit, University of Aberdeen, Aberdeen, UK; 3 Scientific Institute for Quality of Healthcare, Radboud University, Nijmegen, The Netherlands; 4 Dutch College of General Practitioners, Utrecht, The Netherlands; 5 Department of Psychology, University of Aberdeen, Aberdeen, UK

## Abstract

**Background:**

Long term management of patients with Type 2 diabetes is well established within Primary Care. However, despite extensive efforts to implement high quality care both service provision and patient health outcomes remain sub-optimal. Several recent studies suggest that psychological theories about individuals' behaviour can provide a valuable framework for understanding generalisable factors underlying health professionals' clinical behaviour. In the context of the team management of chronic disease such as diabetes, however, the application of such models is less well established. The aim of this study was to identify motivational factors underlying health professional teams' clinical management of diabetes using a psychological model of human behaviour.

**Methods:**

A predictive questionnaire based on the Theory of Planned Behaviour (TPB) investigated health professionals' (HPs') cognitions (e.g., beliefs, attitudes and intentions) about the provision of two aspects of care for patients with diabetes: prescribing statins and inspecting feet.

General practitioners and practice nurses in England and the Netherlands completed parallel questionnaires, cross-validated for equivalence in English and Dutch. Behavioural data were practice-level patient-reported rates of foot examination and use of statin medication. Relationships between the cognitive antecedents of behaviour proposed by the TPB and healthcare teams' clinical behaviour were explored using multiple regression.

**Results:**

In both countries, attitude and subjective norm were important predictors of health professionals' intention to inspect feet (Attitude: beta = .40; Subjective Norm: beta = .28; Adjusted R^2 ^= .34, p < 0.01), and their intention to prescribe statins (Attitude: beta = .44; Adjusted R^2 ^= .40, p < 0.01). Individuals' self-reported intention did not predict practice-level performance of either clinical behaviour.

**Conclusion:**

Using the TPB, we identified modifiable factors underlying health professionals' intentions to perform two clinical behaviours, providing a rationale for the development of targeted interventions. However, we did not observe a relationship between health professionals' intentions and our proxy measure of team behaviour. Significant methodological issues were highlighted concerning the use of models of individual behaviour to explain behaviours performed by teams. In order to investigate clinical behaviours performed by teams it may be necessary to develop measures that reflect the collective cognitions of the members of the team to facilitate the application of these theoretical models to team behaviours.

## Background

Long term management of patients with Type 2 diabetes is now well established within Primary Care. The shift in the provision of care from secondary care has been accompanied by the development of a variety of quality improvement strategies, such as the development and dissemination of evidence-based guidelines and the utilisation of disease management programs [1]. There is a broad international consensus about what constitutes high quality care for people with diabetes [2-4]. However, despite extensive efforts to implement high quality care [5] both service provision and patient health outcomes remain sub-optimal [6].

Systematic reviews have demonstrated that a range of different intervention strategies to enhance diabetes care produce small to modest improvements in glycaemic control and changes in provider behaviour [5,6]. This is also true for interventions across a range of other medical conditions [7,8]. Whilst these findings are encouraging it is less clear how to achieve such change reliably as heterogeneity in study design and setting, and the multi-faceted nature of many interventions makes it difficult to generalise intervention strategies across clinical settings and/or types of health professional. The findings of several recent empirical studies suggest that psychological theories of behaviour can provide a valuable framework for understanding generalisable factors underlying the clinical behaviour of individual health professionals [9-15]. This has paved the way for the development of interventions that target key behavioural processes that are supported by a grounded, empirically tested, scientific rationale [16-18].

**Figure 1:**
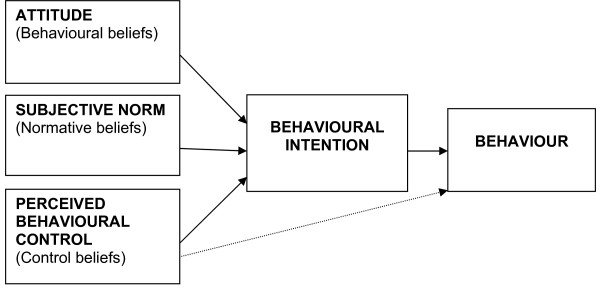
**The Theory of Planned Behaviour (Ajzen, 1991)**. Note. The three variables also influence one another. Although this figure is presented in a simplified form, a more detailed diagram would include double-ended arrows joining these three variables.

One of the more widely used and well tested psychological models is the Theory of Planned Behaviour (TPB) [19]. Like many social cognitive models, the TPB is based on the premise that the way people think influences what they do (i.e. that cognitions, such as beliefs and expectations, influence behaviour). It proposes a model about how human action is guided (Figure 1) which predicts the occurrence of a specific behaviour where a person has an intention to perform that behaviour. According to the TPB, specific behaviours can be predicted by the strength of an individual's intention to enact that behaviour. Intentions are thus the precursors of behaviour and the stronger the intention, the more likely it is that the behaviour will occur. Intention is, in turn, influenced by the individual's attitude towards the behaviour; their perceptions of social pressure to perform the behaviour ("subjective norm"); and the extent to which they feel able to perform the behaviour ("perceived behavioural control"). These latter global constructs are mediated through intention, with only perceived behavioural control (PBC) having a possible direct effect on behaviour.

Previous studies conducted in the primary care setting that have used this approach have usually focused on relatively simple behaviours in the context of the management of a single acute condition (e.g. [11,13,14]). In such contexts, it is the actions of one individual that contribute to the subsequent management of the presenting acute condition (e.g. the prescribing of an antibiotic for sore throat). In the context of chronic disease management, however, the application of models of individual behaviour, such as the TPB, are more challenging. This is because there are several different clinical aspects to the management of diabetes, and the behaviours involved in delivering care are usually shared and delivered by a team rather than by one individual. Different groups of healthcare professionals within a team may also have different, but shared roles and responsibilities (e.g. prescribing may be the sole domain of GPs; foot inspection may be the sole domain of nurses). Alternatively, there may be a specific individual within a team or professional group whose role it is to manage a specific aspect of a patient's care. Thus each aspect of diabetes management may frequently involve not only the actions of more than one healthcare professional but that of different types of healthcare professional. So whilst routinely available data on the quality of care that patients receive within a primary care practice will indicate that a clinical action has been performed, it may not be possible to identify which individual team member performed it, or the data may be a reflection of the collective actions of several team members.

This presents a significant methodological challenge to the use of models of individual behaviour as explanatory frameworks of clinical behaviours performed by teams as they are not normally used in this context. Thus the application of models like the TPB to team behaviours may require an extension of the model and possible elaboration of the methods used to investigate its predictive value.

The current study used the Theory of Planned Behaviour to identify the cognitions of health care professionals', working within primary care clinical teams, about the management of patients with diabetes. In addition to being one of the more widely tested theories in non-clinical populations, this model was chosen because it has been shown to be able to predict healthcare professionals' clinical behaviour [9,20]. Furthermore, clinical behaviour is performed within the current ethos of patient-centred care and in the context of situational constraints such as time pressures. The theoretical constructs in the model appear well placed to take these issues into account. Specifically, Subjective norm (e.g., pressures associated with patient preference) and PBC (whether the clinician has full control over performing all the appropriate behaviours) are proposed to work with Attitude (i.e., the individual's overall evaluation of the behaviour, arising from perceptions of its advantages and disadvantages) to predict intention. Intention predicts behaviour but, within the TPB, the relationship between these two is proposed to be imperfect, with PBC as an effect modifier. The cognitions of interest were those that underlie the management of two key aspects of diabetes care; foot examination (predominantly a nurse or health care assistant behaviour) and the prescribing of statins (a GP behaviour).

To address the methodological issue of relating quality of care data that represent collective behaviours to individual cognitions, the study further explored the relationship between individual cognitions and an independent, practice-level measure of the health care teams' performance in relation to these two clinical behaviours.

### Research questions

Can the TPB predict:

a) the intention of health care professionals to provide two aspects of diabetes care?

b) the teams' collective clinical behaviour in relation to two aspects of diabetes care?

## Methods

### Design and participants

This was a cross-sectional postal survey of primary care health professionals in two European countries. Using a theory-based questionnaire, the study formed part of a process evaluation and was conducted alongside two randomised controlled trials of different interventions to improve the management of patients with diabetes [21,22]. Participants in the study were general practitioners (GPs), practice nurses and assistants, from general practices that were participating in each of the two randomised controlled trials. In the English trial, practices were those recruited to a trial of an "extended" computerised diabetes register that incorporated a structured recall and management system [21]. In the Netherlands practices were those recruited into the PAS trial (The diabetes **P**assport as an **A**id to **S**tructure diabetes management in Primary Care) [22]. Adult patients with type 2 diabetes and receiving care from participating trial practices were also invited to take part in postal questionnaire survey asking about the treatment they had received at their general practice during previous months. In English practices, only patients over the age of 35 years were included and approximately 20% received both GP and specialist care. In Dutch practices patients over the age of 80 years were excluded from participation in the survey, as were patients who received their diabetes treatment in secondary care. English practices were situated in three Primary Care Trusts (PCTs) in the north east of England, served by two district hospital-based diabetes registers. Dutch practices were situated in the middle and south regions of the Netherlands. Both trials reported positive effects of their respective interventions.

### Questionnaires

This study used the TPB in the design of a postal questionnaire survey of healthcare professionals. Four theoretically-derived measures were developed, using the standard procedures recommended for TPB studies [23], to explore: health professionals' intentions to perform each behaviour (e.g. I intend to inspect the feet of patients with diabetes who I see during the next month), their attitude towards it (e.g. Overall I think prescribing statins to patients with diabetes is beneficial to them), their beliefs about perceived social pressure to perform them ("subjective norm", e.g. People who are important to me think that I should inspect the feet of patients with diabetes) and their perceived control over the behaviours (e.g. Prescribing statins to patients with diabetes is easy). As nurses and health care assistants do not routinely prescribe statins they were only asked about foot examination in the final questionnaire. The response format for all items was a seven point Likert-type scale, from 1 (strongly agree) to 7 (strongly disagree). Scores on individual items were averaged to produce a composite measure for each construct, with scores reversed so that a high summary score always indicated stronger or more positive beliefs. The questionnaire was pre-tested with six English GPs and the final version cross-validated to ensure theoretical fidelity. Cross-validation was done by both English and Dutch experts for equivalence in English and Dutch languages using translation (from English to Dutch, by a bilingual researcher who understood the theoretical constructs) and back-translation (from Dutch to English) by a second bilingual researcher (MB). Discrepancies between the original questionnaire items and the back-translation were identified (by JF) and resolved by discussion with a third bilingual researcher. Copies of the English and Dutch versions of the questionnaire are provided in Additional files 1 and 2 respectively.

### Data collection

In both countries the TPB questionnaire was mailed to a total of 220 GPs (161 in England and 59 in the Netherlands) and 141 practice nurses and assistants (119 in England and 22 in the Netherlands) at participating trial practices. Participants were also provided with information about the study and what taking part involved. In accordance with ethical approvals for both trials, consent to participate was given by the return of a completed questionnaire. English non-responders received two reminder letters at fortnightly intervals. Dutch non-responders received one reminder letter after 3 weeks.

Theory-based questionnaire data were collected at the end of the intervention period for both studies (Table 1). Patient questionnaires were also mailed to 4247 patients in both countries at the end of the intervention period (2815 in England and 1432 in the Netherlands). Patients were asked to report what medication they were currently taking and whether or not they had had a foot examination in the past 12 (England) or 15 (Netherlands) months. These patient-reported data were used as a proxy measure of healthcare teams' performance of two clinical behaviours.

**Table 1:** Characteristics of sample and questionnaire response rates from healthcare professionals for the two behaviours.

		Health Professionals		Practices			Median (Interquartile range) per practice	
Overall		GPs	Nurses	Overall	Single GP	>1 GP	GPs	Nurses

Numbers	England	161	119	58	15	43	2 (2)	2 (2)
	
	Netherlands	59	22*	40	15	25	2 (2)	2 (2)
	
	Total	220	141	98	30	68	2 (2)	2 (2)

Response rates (n (%))		GPs	Nurses	Overall	Single GP	>1 GP	GPs	Nurses

Statin prescription	England	59 (37)	-	34 (57)	7 (21)	27 (79)	2 (2)	-
	
	Netherlands	46 (78)	-	35 (88)	11 (31)	24 (69)	3 (2)	-
	
	Total	105 (48)	-	69 (70)	18 (25)	51 (74)	2 (2)	-

Foot examination	England	59 (37)	51 (43)	46 (79)	10 (22)	36 (78)	1 (1)	1 (1)
	
	Netherlands	46 (78)	19** (86)	37 (93)	13 (35)	24 (65)	1 (1)	0 (1)
	
	Total	105 (48)	70 (50)	83 (85)	23 (28)	60 (72)	1 (1)	1 (1)

*Includes 8 nurses and 14 assistants who inspect feet; excludes 26 assistants who did not inspect feet.

**Includes 7 nurses and 12 assistants who inspect feet.

### Statistical analyses

The internal consistency of multi-items measures was assessed using Cronbach's alpha (for measures with three items) and Pearson's correlation coefficient (for measures with two items), using an acceptability criterion of α > 0.6, and r > 0.25 respectively.

Though we have previously shown that predictors of intention differed by trial group within the English study [24] we found no evidence of a trial group effect on intention or behaviour, Data were therefore analysed as two cross sectional studies by pooling the data from trial intervention and control arms within each country. Each study was individually powered to answer a specific set of research questions. One of the aims of the pooling the data in this analysis was so that we could formally compare the results from the two countries. This involved comparing of group of 46 with a group of 69 practices for the prescription of statins and comparing a group of 65 with a group of 110 practices for the recording of feet inspections. These sample sizes gave us 80% power to detect a strength of correlation between two variables (Pearson product moment correlation coefficient) of 0.27 (UK sample), 0.34 (NL sample) 0.21 (combined sample) respectively for the recording of foot inspections and 0.33 (UK sample), 0.40 (NL sample) 0.27 (combined sample) respectively for the prescription of statins, assuming a type 1 error rate of 5%.

It was not possible to attribute patient-reported outcomes to individual health care professionals so these behavioural data were aggregated to the team level. This aggregated variable was the percentage of patients reporting foot examinations or statin use for each general practice. Within each practice, individual health professionals were assigned the aggregated variable for each of the two behaviours. Planned analyses explored the predictive value of the TPB model in explaining variance in health professionals' intention and their assigned behaviour scores. Relationships between the antecedents of intention (attitude, subjective norm and perceived behavioural control) and intention and between intention and clinical behaviour for both foot examination and the prescribing of statins were examined using correlation and multiple regression analysis. As the TPB allows for a direct effect of perceived behavioural control (PBC) on behaviour, PBC was included in the models predicting behaviour. An interaction term was fitted to test for a country effect in all the regression analyses. As both host studies were randomised controlled trials interaction terms were fit into a regression model to test for any respective trial effects on the outcome variables. The appropriateness of regression models was assessed by examining plots of residuals.

Non-response comparisons of practice size (the number of GPs and nurses per practice) were made using Pearson's Chi-square.

### Ethics approval

The studies were conducted in accordance with the tenets of the Declaration of Helsinki and were approved by South Tyneside, Southwest Durham, Hartlepool and North Tees Local Research Ethics Committees in England and the ethics committee of Radboud University Medical centre, Nijmegen, The Netherlands.

## Results

Participant characteristics and survey response rates are shown in Table 1. Two Dutch GPs gave incomplete responses so were excluded from the analysis. The 69 practices contributing at least one GP responder to the statin use analysis were not significantly different in terms of practice size to non-responder practices (Pearson χ^2 ^= 2.248, df = 1, p = 0.13). The 83 practices contributing at least one responder (GP or nurse) to the foot inspection analysis were not significantly different in terms of the number of GPs in the practice (Pearson χ^2 ^= 2.149, df = 1, p = 0.14); but were significantly more likely to have two or more nurses (80% v 47%, Pearson χ^2 ^= 7.215, df = 1, p = 0.007). The English sample had proportionately more nurse respondents (46% v 29%, Pearson χ^2 ^= 4.997, df = 1, p = 0.025).

Usable responses were received from 1433/2815 (51%) English patients and from 993/1432 (69%) Dutch patients. Overall, 736/2426 (30%) patients reported taking statins (362/1433 (25%) English patients and 374/993 (38%) Dutch patients) and 1234/2426 (51%) reported a foot examination (806/1395 (58%) English patients and 428/993 (43%) Dutch patients).

Internal consistency of the TPB measures for both behaviours was satisfactory: Foot examination: Cronbach's alpha: Intention (3 items) = 0.96; Attitude (3 items) = 0.91; Pearson correlation coefficient: Subjective Norm (2 items) = 0.447, p < 0.001); PBC (2 items) = 0.435, p < 0.001); Prescribing statins: Cronbach's alpha: Intention = 0.98; Attitude = 0.95; Pearson Correlation Coefficient: Subjective Norm = 0.564, p < 0.001; PBC = 0.564, p < 0.001). Residual plots suggested that the use of normal regression procedures was appropriate.

Mean scores on the TPB cognitive variables, correlations and rates of patient-reported foot examination and patient-reported statin use are shown in Table 2, for both countries.

**Table 2:** Means and correlations for TPB constructs and two diabetes related clinical behaviours.

	**Foot inspection** (1 = strong disagreement; 7 = strong agreement).					**Prescribing Statins** (1 = strong disagreement; 7 = strong agreement).				
**Country**	INT	ATT	SN	PBC	% Patients reporting foot inspection	INT	ATT	SN	PBC	% Patients reporting statin use

**Netherlands** **(n = 65)**										
Mean (SD)	4.48 (1.89)	6.14 (0.79)	5.23 (1.28)	5.44 (1.18)	39.5 (23)	5.57 (1.36)	6.35 (0.75)	5.29 (1.51)	5.98 (0.98)	37.8 (17)
Pearson Correlation										
*Intention*	-	.36^ns^	.22^ns^	.01^ns^	-.103^ns^	-	.65**	.37*	.30*	.17^ns^
*PBC*	-	-	-	-	.037^ns^	-	-	-	-	.07^ns^

**England** **(n = 110)**										
Mean (SD)	4.69 (1.85)	5.96 (0.99)	5.18 (1.44)	4.73 (1.38)	56.9 (17)	4.65 (1.71)	5.72 (1.18)	5.49 (1.16)	5.65 (1.12)	25.4 (10)
Pearson Correlation										
*Intention*	-	.63**	.61**	.22*	-.135^ns^	-	.57**	.50**	.53**	-.11^ns^
*PBC*	-	-	-	-	-.139^ns^	-	-	-	-	-.09^ns^

**Overall** **(n = 175)**										
Mean (SD)	4.61 (1.86)	6.03 (0.93)	5.20 (1.38)	4.99 (1.35)	49.1 (22)	5.04 (1.63)	6.00 (1.02)	5.40 (1.31)	5.79 (1.07)	31.7 (15)
Pearson Correlation										
*Intention*	-	.53**	.47**	.13^ns^	-.002^ns^	-	.63**	.39**	.48**	.15^ns^
*PBC*	-	-	-	-	-.144^ns^	-	-	-	-	.06^ns^

**. correlation significant at the 0.01 level (2-tailed) *. correlation significant at the 0.05 level (2-tailed) ^ns ^model not significant

INT = Intention. ATT = Attitude. SN = Subjective Norm. PBC = Perceived Behavioural Control

### Foot examination

The intention, subjective norm and attitude scores of health professionals were similar for both countries. Dutch health professionals reported significantly higher perceived behavioural control over foot inspection (Mean difference (95% CI) = 0.71 (0.30 to 1.11), t = 3.441, df_173_, p = 0.001). English patients were significantly more likely to report having had their feet inspected (mean difference (95% CI) 0.17 (0.08 to 0.26), t = 3.372, df_81_, p < 0.001).

#### Predicting intention (individual-level outcome variable)

Attitude, subjective norm and PBC were regressed on intention to inspect feet (Table 3, Model 1). Attitude significantly predicted intention to inspect patients' feet for both English and Dutch health professionals. Subjective norm significantly predicted intention for Dutch health professionals; no significant interaction was found between country and subjective norm (β = -.286, p = 0.117), indicating that there is no difference in the importance of this variable between the two countries. There was no main effect for Country in this model. Together attitude and subjective norm explained approximately 34% of the variance observed in health professionals' reported intention to inspect feet.

**Table 3:** Regression models for TPB constructs and two diabetes related clinical behaviours, by country and overall.

**Behaviour**	**Foot inspection**			**Prescribing Statins**		
	**Netherlands** **(n = 65)**	**England** **(n = 110)**	**Overall** **(n = 175)**	**Netherlands** **(n = 46)**	**England** **(n = 69)**	**Overall** **(n = 105)**
**Model**	**Standardised *β*** **Adj R^2^**	**Standardised *β*** **Adj R^2^**	**Standardised *β*** **Adj R^2^**	**Standardised *β*** **Adj R^2^**	**Standardised *β*** **Adj R^2^**	**Standardised *β*** **Adj R^2^**

**1:Predicting** **Intention**						
*Attitude*	.34**	.41**	.40**	.64**	.29^ns^	.44**
*SN*	.15^ns^	.37**	.28**	.09^ns^	.18^ns^	.12^ns^
*PBC*	-.09^ns^	.07^ns^	.02^ns^	-.04^ns^	.25^ns^	.14^ns^
*Country*	-	-	.10^ns^	-	-	.13^ns^
***Adjusted R*^2^**	.11*	.48**	.34**	.39**	.35**	.40**

**2: Predicting** **Behaviour**						
*Intention*	.103^ns^	-.109^ns^	-.01^ns^	16^ns^	-.09^ns^	.04^ns^
*PBC*	.036^ns^	-.115^ns^	-.06^ns^	.03^ns^	-.04^ns^	-.02^ns^
*Country*	-	-	.33**	-	-	.40**
***Adjusted R*^2^**	-.02^ns^	.01^ns^	.11**	-.02^ns^	.02^ns^	.15**

**. model significant at the 0.01 level *. model significant at the 0.05 level, ^ns ^model not significant

#### Predicting behaviour (team-level outcome variable)

Intention and PBC were regressed on behaviour (Table 3, Model 2). Neither intention nor PBC predicted foot inspection behaviour. As there was a significant difference in mean rates of patient reported foot inspection between the two countries, a "country" variable was allowed into the overall model. An interaction term was also fitted to formally test the relationship between PBC and country. The interaction was non-significant (β = .022, p = 0.343). There was a significant main effect of Country.

### Prescribing Statins

Scores for subjective norm and perceived behavioural control over prescribing of statins of GPs were similar for both countries. While the overall strength of GP intention to prescribe statins and their attitude towards this behaviour were high, Dutch GPs reported significantly more positive intention and attitudes towards prescribing statins (Mean difference (95% CI): Intention = 0.919 (0.30 to 1.54), t = 2.933, df_101_, p = 0.004; Attitude = 0.641 (0.26 to 1.02), t = 3.343, df_103_, p = 0.001). In English practices the mean (sd) percentage of patients who reported taking a statin was 25.4 (10)% and in Dutch practices this was 37.7 (16.6)% (mean difference (95% CI) 0.123 (0.06 to 0.19), t = 3.749, df_67_, p < 0.001).

#### Predicting intention (individual-level outcome variable)

Attitude, subjective norm and PBC were regressed on intention to prescribe statins (Table 3, Model 1). Attitude significantly predicted intention for Dutch GPs. However, no significant interaction was found between country and attitude (β = .280, p = 0.347). There was no other apparent country effect. GPs' attitudes towards prescribing statins explained approximately 40% of the variance observed in their reported intention to perform this behaviour.

#### Predicting behaviour (team-level outcome variable)

Intention and PBC were regressed on behaviour (Table 3, Model 2). Neither intention nor PBC predicted statin prescribing behaviour. As there was a significant difference in mean rates of patient-reported statin use between the two countries, a "country" variable was also allowed into the overall model. An interaction term was also fitted to formally test the relationship between PBC and country. The interaction was non-significant (β = -2.259, p = 0.402) indicating that the relationship between PBC and prescribing behaviour did not differ between countries. There remained a significant country effect not explained by the TPB constructs.

## Discussion

This study has shown that the variables specified by the Theory of Planned Behaviour were important predictors of health professionals' intention to inspect feet and to prescribe statins. Primary Care health professionals' attitudes towards both the clinical behaviours investigated and their perceived social pressure to perform them accounted for a significant amount of the variance in their intention to provide these elements of diabetes care. This was found to be true in general for health professionals from two European countries in relation to inspecting the feet of diabetic patients. However, we did not find a relationship between health professionals' intention, or their perceived behavioural control measured at the individual level and our patient-reported measure of behaviour (which reflected team-level behaviour). This is despite the findings of two recent systematic reviews suggesting that social cognition models of behaviour, which have been successfully used to predict behaviour and behavioural change in non-clinical populations, can be usefully applied to clinical behaviour at the individual level [9,20].

This difference between the results of individual level studies and the present study predicting team behaviours may result from lack of correspondence between the measures of cognitions and behaviours. Fundamental to the Theory of Planned Behaviour is Fishbein's "TACT" principle of correspondence [25]; which is that measures of intention and behaviour must be specified at the same level of generality. Measures correspond if they relate to the same operational definitions of the: ***T**arget *of the action (in the present study this is any patient with type 2 diabetes); ***A**ction *to be performed (e.g. foot examination); ***C**ontext *in which the action is performed (e.g. during a consultation) and the specified ***T**ime *period (e.g. over the next/last month).

For foot examination, the measures used in the present study to assess this behaviour adhered closely to this "TACT" principle in that the wording of the questionnaire items in our patient-reported measure corresponded closely to those in the health professional measure. Thus it is unlikely that poor correspondence between the wording of these measures used to quantify intention and behaviour for foot examination contributed to error [9]. For the prescribing of statins, however, one question in the patient report measure may have been too general; rather than ask patients if they had been prescribed statins we asked them to list all the medication they had taken in the past 4 weeks. Wording the question this way changed the focus of whose behaviour we were asking about (and reduced the specificity of the *Action*), potentially introducing some non-reporting of statin use that reflected patient non-compliance and/or recall bias. Future attempts to use individual level theories such as the TPB in the context of behaviours delivered by clinical teams should address the problem of correspondence by seeking alternative methods of measuring or aggregating cognitions about the clinical behaviour as well as improving the measures of clinical behaviour.

The ability of social cognition theories like TPB to predict clinicians' behaviour has been demonstrated in studies using both self-reported and objective (observed) measures (varying between 13% [20] and 20% [26] for objective measures), though the amount of variance in behaviour that is explained by such models is consistently lower when an objective measure of clinician behaviour (like patient report) is obtained [9,26]. There are several factors that could account for the finding that social cognitive models predict intention more strongly than they predict behaviour. Among them is the "intention-behaviour" gap. There is a considerable literature that addresses this gap (e.g. [27]) which highlights the importance of "post-intentional" factors that intervene to mediate an individual's behaviour, given the existence of a strong intention. However, while it is highly possible that such factors contributed to the findings presented here, an alternative explanation for the lack of an observed association between intention and behaviour in the present study could be a lack of "correspondence" between individuals' cognitions and the aggregated measure of behaviour that we used. i.e., the predictors (including intention) were measured at the level of the individual clinician and behaviour was measured at the practice level.

This latter explanation presents a methodological challenge to the use of social cognitive models to investigate clinical behaviours as it is not always possible to achieve such a precise link between the measures of cognition and behaviour. This is a problem which is amplified in the investigation of behaviours that are performed within the context of a team; some behaviours may be shared (e.g. foot inspection may be the role of more than one nurse or health care assistant and the prescribing of statins the role of more than one GP) and others may contribute cumulatively to a single aspect of care (e.g. in the weight management of people with diabetes a nurse may provide lifestyle counselling, a dietician give dietary advice and a GP prescribe a weight loss medication).

Hence for the behaviours investigated in the present study it was not possible to link the measures of intention and behaviour so precisely. Instead, patient-reported rates of statin use and foot inspection were aggregated to practice level and the mean value assigned to individual health professionals within each practice. This strategy assumes that each health professional has an equal role in the performance of the behaviour of interest – i.e. that the behaviour is a shared role. Where this is not the case – when for example a single GP takes the lead in providing care for patients with diabetes in one practice, or it is the role of a single nurse to examine patients' feet – this strategy reduces the specificity of this measure of behaviour. Further more, the latter scenario would not necessarily result in other team members having less favourable attitudes etc towards the clinical behaviours investigated here. They may, however, have little or no intention to perform those behaviours because they are confident that these actions will be covered by other members of the clinical team, reducing the ability of this measure to predict behaviour. Thus some alternative methods of aggregating the collective cognitions of the team might lead to stronger prediction of the collective behaviour.

There are additional problems in the measurement of the clinical behaviours. We used patient reported measures as these were the only measures in common for these behaviours across the two host trials. While patient- and self-report measures are commonly used as proxies for actual behaviour in implementation research, these, along with other frequently used proxy measurement methods, do have limitations which can threaten their validity. The patient data used in the present study may have been biased by the low response rates to the patient survey; while 69% of useable responses were obtained for the Dutch patient questionnaire only 51% were obtained for the English patient sample. In addition, we did not have sufficient information about the approached samples that would allow further evaluation of how representative those responding were of the respective patient populations. Encouragingly, the rates of statin use and foot inspection reported by the English patients in this study are supported by additional data from medical records reported elsewhere [28]. Data from this addition source suggest that there was no difference in levels of clinician performance as reported in the adjusted record based data and the unadjusted patient-report based data. This provides some evidence that these proxy measures may provide an adequate measure of actual rates of statin prescription and foot inspection.

### Limitations

This study is limited by the low response rate to the English survey [24]. This was particularly low at the individual level for both behaviours (37%), but improved at practice level (statin use 57%, foot inspection 79%). This may have been due to greater respondent burden for the English HPs as the English survey instrument consisted 154 items and covered three behaviours. However, while non-response analysis indicated that nurse respondents were over-represented in the English dataset, both the English and the Dutch practices responding to the surveys were largely representative of practices enrolled on the two trials.

The psychological model we used relates to the intentions and behaviour of individuals but the two aspects of diabetes care that we examined are performed in the context of the team management of this chronic disease. As we did not survey all practice staff within each participating practice, it is feasible that the cognitions of key health professionals whose role involved providing the behaviours of interest were not included in the study. It is also possible that either one or both of the behaviours measured were not performed by all health professionals who did respond to the survey. Allocating our aggregated measure of behaviour to these respondents assumed that they had. A further limitation may have been our use of an acceptability criterion of r > 0.25 for internal consistency for 2-item measures. However our 2-item measures were found to be well above this minimum threshold. These methodological limitations had the potential to reduce the correspondence of the measures used and thus the predictive ability of the TPB model to explain clinicians' behaviour in the context of a team setting.

## Conclusion

The findings of this study are very exploratory in nature and suggest associations rather than causes. Despite its limitations however, this study has identified modifiable factors underlying health professionals' intentions to perform two clinical behaviours, providing a rationale for the development of targeted interventions. This study adds to the growing body of evidence that psychological models of human behaviour may be of value in the prediction of health professionals' intentions to perform clinical behaviours. However, we did not observe a relationship between health professionals' intentions and our proxy measure of team behaviour. Importantly, the study also highlights significant methodological challenges to the use of social cognitive models of individual behaviour to explain behaviours performed as part of the team management of chronic diseases like diabetes.

The lack of a direct link between individuals' cognitions and behaviour compromised the correspondence between measures (a fundamental feature of the TPB) and may explain the lack of association between intention and behaviour. However, in order to use a theory-based approach to behaviours that are performed in the context of a team – such as diabetes care – it may be necessary to develop the measurement of the theoretical constructs to facilitate their application to team behaviours. It may, for example, be necessary to consider different strategies for aggregating scores that represent individuals' cognitions when their collective behaviours contribute to a single outcome. This is the subject of a separate methodological paper by the authors.

## Competing interests

The authors declare that they have no competing interests.

## Authors' contributions

The study was conceived and designed by MPE, JF, MB, RD, MJ, RG, ESFK and INS. INS and SH performed the analysis. Writing of the manuscript was led by SH. All authors commented on all drafts and approved the final version.

## Pre-publication history

The pre-publication history for this paper can be accessed here:



## Supplementary Material

Additional file 1 **UK Clinician survey**. Theory-based survey instrument English language. Click here for file

Additional file 2 **ND Clinician survey**. Theory-based survey instrument Dutch language. Click here for file
