# Supplementary material for: Using psychological theory to understand the clinical management of type 2 diabetes in Primary Care: a comparison across two European countries
Source: BMC Health Serv Res. 2009 Aug 5;9:140. doi: 10.1186/1472-6963-9-140 (PMC2729744; doi:10.1186/1472-6963-9-140)
Supplement: Additional file 1 — UK Clinician survey. Theory-based survey instrument English language. [file 1472-6963-9-140-S1.pdf]

About your BACKGROUND

1. How long have you been qualified?	☐☐	**Years**
2. How many sessions (1/2 days) do you work per week?	☐☐	
3. How many GPs work at your practice?	☐☐	
4. How many practice nurses work at your practice?	☐☐	
5. Are you **Male** ☐	Or **Female** ☐	
6. Are you a **GP** ☐	Or a **Practice Nurse** ☐	

If you are a GP please go to Question 10. If you are a Practice Nurse please answer questions 7-9

7. If you are a nurse do you provide care for people with Type 2 diabetes?	**Yes** ☐	**No** ☐
8. If you are a nurse do you prescribe?	**Yes** ☐	**No** ☐
9. If Yes, do you prescribe statins for people you see with Type 2 diabetes?	**Yes** ☐	**No** ☐
10. What is your approximate practice list size?	☐☐☐☐☐	

Each question in the following section refers to MEASURING THE BLOOD PRESSURE of your patients with Type 2 diabetes.

Please circle your answer

		Strongly Agree						*Strongly Disagree*
1.	If I measure their blood pressure, I feel that I am doing something positive for the patient	1	2	3	4	5	6	7
2.	If I measure a patient's blood pressure, I will detect any problems at an early stage	1	2	3	4	5	6	7
3.	If I measure a patient's blood pressure, I will identify those at risk and treat them appropriately	1	2	3	4	5	6	7
4.	It causes a lot of worry and concern for the patient if they are found to have high blood pressure	1	2	3	4	5	6	7
5.	Patients can get very upset if I measure their blood pressure	1	2	3	4	5	6	7
6.	If I measure their blood pressure, I've got to see some patients more often	1	2	3	4	5	6	7
7.	Doing something positive for the patient is very important to me	1	2	3	4	5	6	7
8.	It is vital not to cause worry and concern in patients if they are found to have high blood pressure	1	2	3	4	5	6	7

		Strongly Agree						*Strongly Disagree*
9.	Patients getting upset is of great concern to me	1	2	3	4	5	6	7
10.	The number of times I see a patient is very important to me	1	2	3	4	5	6	7
11.	Other GPs think I should measure the blood pressure of patients with diabetes	1	2	3	4	5	6	7
12.	Diabetologists would approve of me measuring the blood pressure of patients with diabetes	1	2	3	4	5	6	7
13.	Patients would approve of me measuring their blood pressure	1	2	3	4	5	6	7
14.	The government would approve of me measuring the blood pressure of patients with diabetes	1	2	3	4	5	6	7
15.	People who think that measuring blood pressure is not a GP's job would disapprove of me doing it	1	2	3	4	5	6	7
16.	The view of people who think that measuring blood pressure is not a GP's job is of importance to me	1	2	3	4	5	6	7
17.	The evidence about treating raised blood pressure in patients with diabetes is unequivocal	1	2	3	4	5	6	7
18.	Sometimes the blood pressure machine I use is not very accurate/does not give a reading every time	1	2	3	4	5	6	7
19.	Sometimes the cuffs on the blood pressure machines are uncomfortable and/or do not fit patients properly	1	2	3	4	5	6	7
20.	Sometimes when I am measuring blood pressure in the consultation I feel rushed	1	2	3	4	5	6	7
21.	Sometimes the patient is inappropriately dressed to have their blood pressure measured	1	2	3	4	5	6	7
22.	Sometimes the patient likes to have their blood pressure checked	1	2	3	4	5	6	7
23.	Some patients don't want to change their lifestyle in order to bring down their blood pressure	1	2	3	4	5	6	7
24.	"White coat syndrome" can make a patient's blood pressure rise	1	2	3	4	5	6	7
25.	Controlling blood pressure is one of the harder diabetes care targets to achieve	1	2	3	4	5	6	7
26.	If the patient has high blood pressure they think they have another illness as well as diabetes	1	2	3	4	5	6	7
27.	The patients do not always believe the blood pressure reading I give them in the consultation	1	2	3	4	5	6	7
28.	I am more likely to measure a patient's blood pressure if the blood pressure machine that I use is accurate and gives a reading every time	1	2	3	4	5	6	7

		Strongly Agree						*Strongly Disagree*
29.	I am more likely to measure a patient's blood pressure if the cuff is comfortable for the patient and fits properly	1	2	3	4	5	6	7
30.	I am more likely to measure a patient's blood pressure if there was more time in consultations	1	2	3	4	5	6	7
31.	I am more likely to measure a patient's blood pressure if BP if they have a short sleeved top on and are not wearing a coat	1	2	3	4	5	6	7
32.	I am more likely to measure a patient's BP if they want to have their blood pressure checked	1	2	3	4	5	6	7
33.	I am more likely to measure a patient's blood pressure if I think they are willing to change their lifestyle	1	2	3	4	5	6	7
34.	I am more likely to check blood pressure if the patient does not get anxious	1	2	3	4	5	6	7
35.	If controlling raised blood pressure was easier to achieve I would be more likely to measure it	1	2	3	4	5	6	7
36.	If the patients did not see raised blood pressure as a separate illness to diabetes I would be more likely to measure their blood pressure	1	2	3	4	5	6	7
37.	If the patients always trusted the blood pressure readings I gave them I would be more likely to measure blood pressure	1	2	3	4	5	6	7
38.	I intend to measure the blood pressure of most of the patients' with diabetes that I see during the next month	1	2	3	4	5	6	7
39.	I expect to measure the blood pressure of most of the patients' with diabetes that I see during the next month	1	2	3	4	5	6	7
40.	I want to measure the blood pressure of most of the patients' with diabetes that I see during the next month	1	2	3	4	5	6	7
41.	Overall I think measuring these patients' blood pressure is beneficial to them	1	2	3	4	5	6	7
42.	Overall I think measuring these patients' blood pressure is the right thing to do	1	2	3	4	5	6	7
43.	Overall I think measuring these patients' blood pressure is good practice	1	2	3	4	5	6	7
44.	People who are important to me think that I should measure the blood pressure of my patients with diabetes	1	2	3	4	5	6	7
45.	My professional body think that I should measure the blood pressure of my patients with diabetes	1	2	3	4	5	6	7
46.	Measuring patients' blood pressure is easy	1	2	3	4	5	6	7
47.	Overall, I feel that I can measure the blood pressure of most of my patients with diabetes in the next month	1	2	3	4	5	6	7

		Please insert number
48.	From memory, approximately how many of the last 10 patients you saw with Type 2 diabetes, did you measure blood pressure of?	
49.	Out of 10 patients with Type 2 Diabetes, how many would you expect to measure blood pressure of?	

Each of the questions in the following section refers to FOOT EXAMINATIONS on your patients with Type 2 diabetes.

Please circle your answer

		Strongly Agree						*Strongly Disagree*
1.	If I ensure that a foot examination is carried out I will catch any problems at an early stage	1	2	3	4	5	6	7
2.	If I ensure that a foot examination is carried out I will reduce the risk of further complications	1	2	3	4	5	6	7
3.	If I do a foot examination, patients could get a false sense of security	1	2	3	4	5	6	7
4.	If a foot examination is carried out the patient will be encouraged to care for their feet	1	2	3	4	5	6	7
5.	Giving patients a false sense of security is a significant issue	1	2	3	4	5	6	7
6.	Getting patients to care for their own feet is very important	1	2	3	4	5	6	7
7.	Podiatrists and chiropodists think I should examine patients' feet	1	2	3	4	5	6	7
8.	Other GPs in my practice think I should examine patients' feet	1	2	3	4	5	6	7
9.	The government thinks I should examine patients' feet	1	2	3	4	5	6	7
10.	My patients think I should examine their feet	1	2	3	4	5	6	7
11.	Diabetologists think that I should examine patients' feet	1	2	3	4	5	6	7
12.	It is important to me that podiatrists and chiropodists approve of my practice	1	2	3	4	5	6	7
13.	Screening the feet of these patients is cost effective	1	2	3	4	5	6	7

		Strongly Agree						Strongly Disagree
14.	Some patients come to the consultation with a foot problem	1	2	3	4	5	6	7
15.	Some patients come to the consultation prepared for a foot examination and appropriately dressed	1	2	3	4	5	6	7
16.	Some patients have poor foot hygiene	1	2	3	4	5	6	7
17.	Examining feet takes up time in the consultation	1	2	3	4	5	6	7
18.	The cost effectiveness of screening feet makes me more likely to do a foot examination	1	2	3	4	5	6	7
19.	I am more likely to do a foot examination if the patient has come in with a foot problem	1	2	3	4	5	6	7
20.	If the patient is not prepared for a foot examination I am less likely to do one at that time	1	2	3	4	5	6	7
21.	Poor foot hygiene makes me less likely to do a foot examination	1	2	3	4	5	6	7
22.	Lack of time in the consultation makes me less likely to do a foot examination	1	2	3	4	5	6	7
23.	I intend to examine the feet of all my patients I see in the next month who have not been examined by the chiropodist or the podiatrist	1	2	3	4	5	6	7
24.	I expect to examine the feet of all my patients I see in the next month who have not been examined by the chiropodist or the podiatrist	1	2	3	4	5	6	7
25.	I want to examine the feet of all my patients I see in the next month who have not been examined by the chiropodist or the podiatrist	1	2	3	4	5	6	7
26.	Overall I think examining patients' feet is beneficial to them	1	2	3	4	5	6	7
27.	Overall I think examining the feet of patients is the right thing to do	1	2	3	4	5	6	7
28.	Overall I think examining the feet of patients is good practice	1	2	3	4	5	6	7
29.	People who are important to me think that I should examine the feet of those patients who have not been examined by the chiropodist or the podiatrist	1	2	3	4	5	6	7
30.	My professional body think that I should examine the feet of those patients who have not been examined by the chiropodist or the podiatrist	1	2	3	4	5	6	7
31.	Examining patients' feet is easy	1	2	3	4	5	6	7
32.	Overall I feel that I can examine these patients' feet if I want to	1	2	3	4	5	6	7

		Please insert Number
33.	From memory, approximately how many of the last 10 patients you saw with Type 2 diabetes, did you do a foot examination for?	
34.	Out of 10 patients with Type 2 Diabetes, how many would you expect to do a foot examination for?	

Each question in the following section refers to the PRESCRIBING OF STATINS to your patients with Type 2 diabetes.

If you are a practice nurse and you do not prescribe statins please try and answer the following questions about prescribing statins by substituting the word 'practice' instead of 'I'. Try to think what the practice as a whole would do and believe

Please circle your answer

		Strongly Agree						*Strongly Disagree*
1.	If I prescribe a statin the patient will benefit because it will lower their cholesterol	1	2	3	4	5	6	7
2.	If I prescribe a statin the patient will benefit from a reduced risk of cardiac events/long term morbidity	1	2	3	4	5	6	7
3.	If I prescribe a statin the patient will have a reduced risk of mortality	1	2	3	4	5	6	7
4.	If I prescribe a statin the patient will only experience mild side effects from it	1	2	3	4	5	6	7
5.	If I prescribe a statin the patient may not take it as recommended	1	2	3	4	5	6	7
6.	If I prescribe a statin it will discourage patients from changing their lifestyle	1	2	3	4	5	6	7
7.	If I prescribe a statin the patient will be on it for the rest of their life	1	2	3	4	5	6	7
8.	Lowering the patient's cholesterol is important	1	2	3	4	5	6	7
9.	Minimising the side effects of drugs is important	1	2	3	4	5	6	7
10.	Non-adherence to drug treatments is an important issue	1	2	3	4	5	6	7
11.	Discouraging patients from changing their lifestyle is harmful	1	2	3	4	5	6	7
12.	Patients having to stay on drugs for the rest of their lives is an important issue	1	2	3	4	5	6	7

		Strongly Agree						*Strongly Disagree*
13.	Other GPs would approve of me prescribing statins to patients with diabetes	1	2	3	4	5	6	7
14.	The government would approve of me prescribing statins to patients with diabetes	1	2	3	4	5	6	7
15.	Patients would approve of me prescribing statins	1	2	3	4	5	6	7
16.	Older patients would disapprove of me prescribing statins	1	2	3	4	5	6	7
17.	Local diabetologists would disapprove of me prescribing statins	1	2	3	4	5	6	7
18.	Older patients' approval of what I do is important to me	1	2	3	4	5	6	7
19.	I would recommend changing diet and lifestyle before prescribing statins	1	2	3	4	5	6	7
20.	Statins are expensive	1	2	3	4	5	6	7
21.	There is much evidence in support of statins in the treatment of diabetes	1	2	3	4	5	6	7
22.	There are a lot of things to remember in the treatment of diabetes	1	2	3	4	5	6	7
23.	Much controversy exists about the use of statins in the treatment of diabetes	1	2	3	4	5	6	7
24.	There is a lack of consensus about who should receive statins and/or the starting dosage	1	2	3	4	5	6	7
25.	I am concerned about the long term side effects of statins	1	2	3	4	5	6	7
26.	Some patients do not want to take statins	1	2	3	4	5	6	7
27.	Patients who are over 75 years of age do not want to take statins	1	2	3	4	5	6	7
28.	I would have to prescribe a lot of statins in order to save one life	1	2	3	4	5	6	7
29.	Patients who have borderline high cholesterol (i.e. on the margin) will not benefit from statins	1	2	3	4	5	6	7
30.	If diet and lifestyle change has failed to reduce cholesterol then I am more likely to prescribe a statin	1	2	3	4	5	6	7
31.	If statins were less expensive I would be more likely to prescribe them	1	2	3	4	5	6	7
32.	The amount of evidence in support of statins in the treatment of diabetes makes me more likely to prescribe them	1	2	3	4	5	6	7

		Strongly Agree						Strongly Disagree
33.	I would be more likely to prescribe statins if there were not so many things to remember in the treatment of diabetes	1	2	3	4	5	6	7
34.	Having to prescribe a lot of statins in order to save one life makes me less likely to prescribe	1	2	3	4	5	6	7
35.	Controversy about the use of statins makes me less likely to prescribe them	1	2	3	4	5	6	7
36.	A lack of consensus about who should receive statins and/or the starting dosage makes me less likely to prescribe them	1	2	3	4	5	6	7
37.	Concerns about the long term side effects of statins makes me less likely to prescribe them	1	2	3	4	5	6	7
38.	I am less likely to prescribe a statin if the patient doesn't want to take them	1	2	3	4	5	6	7
39.	I am less likely to prescribe a statin if the patient has borderline high cholesterol (i.e. on the margin)	1	2	3	4	5	6	7
40.	I am less likely to prescribe a statin if the patient is over 75 years of age	1	2	3	4	5	6	7
41.	I intend to prescribe a statin to most of the patients I see in the next month	1	2	3	4	5	6	7
42.	I expect to prescribe a statin to most of the patients I see in the next month	1	2	3	4	5	6	7
43.	I want to prescribe a statin to most of the patients I see in the next month	1	2	3	4	5	6	7
44.	Overall I think prescribing a statin to patients is beneficial to them	1	2	3	4	5	6	7
45.	Overall I think prescribing a statin to patients is the right thing to do	1	2	3	4	5	6	7
46.	Overall I think prescribing a statin to patients is good practice	1	2	3	4	5	6	7
47.	People who are important to me think that I should prescribe statins to these patients	1	2	3	4	5	6	7
48.	My professional body thinks that I should prescribe statins to patients	1	2	3	4	5	6	7
49.	To prescribe a statin is easy	1	2	3	4	5	6	7
50.	Overall, I feel that I can prescribe statins if I want to	1	2	3	4	5	6	7

		Please insert a number
51.	From memory, approximately how many of the last 10 patients you saw with Type 2 diabetes, did prescribe a statin for?	
52.	Out of 10 patients with Type 2 Diabetes, how many would you expect to prescribe a statin for?	

This final section asks about your GENERAL BELIEFS about the treatment of Type 2 diabetes.

Please circle your answer

		Strongly Agree						*Strongly Disagree*
1.	Detecting any problems at an early stage is very important	1	2	3	4	5	6	7
2.	Identifying those at risk and treating them appropriately is very important	1	2	3	4	5	6	7
3.	What other GPs think I should do matters to me	1	2	3	4	5	6	7
4.	What Diabetologists think I should do matters to me	1	2	3	4	5	6	7
5.	Patients' approval of my practice is of importance to me	1	2	3	4	5	6	7
6.	The government's approval of what I do is of importance to me	1	2	3	4	5	6	7
7.	I am more likely to do something that is supported by research evidence	1	2	3	4	5	6	7

8. Do you have a clear idea how you would want to manage patients with Type 2 diabetes?

Yes ☐ **No** ☐ **Unsure** ☐

9. If Yes, please describe the main steps briefly ______________________
