# Supplementary material for: Using psychological theory to understand the clinical management of type 2 diabetes in Primary Care: a comparison across two European countries
Source: BMC Health Serv Res. 2009 Aug 5;9:140. doi: 10.1186/1472-6963-9-140 (PMC2729744; doi:10.1186/1472-6963-9-140)
Supplement: Additional file 2 — ND Clinician survey. Theory-based survey instrument Dutch language. [file 1472-6963-9-140-S2.pdf]

SECTIE 1

Elke vraag in deze sectie heeft betrekking op de behandeling van uw patiënten met Type 2 diabetes. Omcirkel voor elke vraag alstublieft telkens één nummer dat het meeste met uw mening overeenkomt, van 1 (geheel mee oneens) tot 7 (geheel mee eens) met de tussenliggende stappen.

		Geheel mee oneens						*Geheel mee eens*
1.	Mensen die belangrijk voor mij zijn vinden dat ik de bloeddruk van mijn diabetespatiënten moet meten	1	2	3	4	5	6	7
2.	Mijn professionele organisatie vindt dat ik de bloeddruk van mijn diabetespatiënten moet meten	1	2	3	4	5	6	7
3.	Het meten van de bloeddruk van mijn diabetespatiënten is moeilijk	1	2	3	4	5	6	7
4.	Ik ben van plan om de bloeddruk te meten van al mijn diabetespatiënten tijdens hun volgende consult	1	2	3	4	5	6	7
5.	Ik verwacht de bloeddruk te meten van al mijn diabetespatiënten tijdens hun volgende consult	1	2	3	4	5	6	7

6.	In het algemeen is volgens mij het meten van de bloeddruk van mijn diabetespatiënten:	*Schadelijk*	1	2	3	4	5	6	7	*Nuttig*
7.		*Prettig*	1	2	3	4	5	6	7	*Niet prettig*
8.		*Verkeerd om te doen*	1	2	3	4	5	6	7	*Juist om te doen*
9.		*Goed handelen*	1	2	3	4	5	6	7	*Slecht handelen*

		Geheel mee oneens						*Geheel mee eens*
10.	Ik wil de bloeddruk meten van al mijn diabetespatiënten tijdens hun volgende consult	1	2	3	4	5	6	7
11.	Andere huisartsen meten de bloeddruk van hun diabetespatiënten niet	1	2	3	4	5	6	7
12.	Of ik de bloeddruk van mijn diabetespatiënten meet is geheel mijn zaak	1	2	3	4	5	6	7
13.	In het algemeen denk ik niet dat ik de bloeddruk van mijn diabetespatiënten kan meten	1	2	3	4	5	6	7

		Geheel mee oneens						*Geheel mee eens*
1.	Mensen die belangrijk voor mij zijn vinden dat ik de voeten van mijn diabetespatiënten moet controleren	1	2	3	4	5	6	7
2.	Mijn professionele organisatie vindt dat ik de voeten van mijn diabetespatiënten moet controleren	1	2	3	4	5	6	7
3.	Het controleren van de voeten van mijn diabetespatiënten is moeilijk	1	2	3	4	5	6	7
4.	Ik ben van plan om de voeten te controleren van al mijn diabetespatiënten tijdens hun volgende consult	1	2	3	4	5	6	7
5.	Ik verwacht de voeten te controleren van al mijn diabetespatiënten tijdens hun volgende consult	1	2	3	4	5	6	7

6.	In het algemeen is volgens mij het controleren van de voeten van mijn diabetespatiënten:	***Schadelijk***	1	2	3	4	5	6	7	***Nuttig***
7.		***Prettig***	1	2	3	4	5	6	7	***Niet prettig***
8.		***Verkeerd om te doen***	1	2	3	4	5	6	7	***Juist om te doen***
9.		***Goed handelen***	1	2	3	4	5	6	7	***Slecht handelen***

		Geheel mee oneens						*Geheel mee eens*
10.	Ik wil de voeten controleren van al mijn diabetespatiënten tijdens hun volgende consult	1	2	3	4	5	6	7
11.	Andere huisartsen controleren de voeten van hun diabetespatiënten niet	1	2	3	4	5	6	7
12.	Of ik de voeten van mijn diabetespatiënten controleer is geheel mijn zaak	1	2	3	4	5	6	7
13.	In het algemeen denk ik niet dat ik de voeten van mijn diabetespatiënten kan controleren	1	2	3	4	5	6	7

		Geheel mee oneens						*Geheel mee eens*
1.	Mensen die belangrijk voor mij zijn vinden dat ik statines moet voorschrijven aan mijn diabetespatiënten	1	2	3	4	5	6	7
2.	Mijn professionele organisatie vindt dat ik statines moet voorschrijven aan mijn diabetespatiënten	1	2	3	4	5	6	7
3.	Het voorschrijven van statines aan mijn diabetespatiënten is moeilijk	1	2	3	4	5	6	7
4.	Ik ben van plan om statines voor te schrijven aan al mijn diabetespatiënten tijdens hun volgende consult	1	2	3	4	5	6	7
5.	Ik verwacht statines voor te schrijven aan al mijn diabetespatiënten tijdens hun volgende consult	1	2	3	4	5	6	7

6.	In het algemeen is volgens mij het voorschrijven van statines aan mijn diabetespatiënten:	***Schadelijk***	1	2	3	4	5	6	7	***Nuttig***
7.		***Prettig***	1	2	3	4	5	6	7	***Niet prettig***
8.		***Verkeerd om te doen***	1	2	3	4	5	6	7	***Juist om te doen***
9.		***Goed handelen***	1	2	3	4	5	6	7	***Slecht handelen***

		Geheel mee oneens						*Geheel mee eens*
10.	Ik wil statines voorschrijven aan al mijn diabetespatiënten tijdens hun volgende consult	1	2	3	4	5	6	7
11.	Andere huisartsen schrijven geen statines voor aan hun diabetespatiënten	1	2	3	4	5	6	7
12.	Of ik statines voorschrijf aan mijn diabetespatiënten is geheel mijn zaak	1	2	3	4	5	6	7
13.	In het algemeen denk ik niet dat ik statines kan voorschrijven aan mijn diabetespatiënten	1	2	3	4	5	6	7
